# Radiological Benefits of Vitamin D Status and Supplementation in Patients with MS—A Two-Year Prospective Observational Cohort Study

**DOI:** 10.3390/nu15061465

**Published:** 2023-03-17

**Authors:** Weronika Galus, Tomasz Chmiela, Anna Walawska-Hrycek, Ewa Krzystanek

**Affiliations:** 1 Department of Neurology, Faculty of Medical Sciences in Katowice, Medical University of Silesia, 40-752 Katowice, Poland; 2 Department of Neurology, Faculty of Health Sciences in Katowice, Medical University of Silesia, 40-635 Katowice, Poland

**Keywords:** vitamin D, multiple sclerosis, vitamin D supplementation

## Abstract

Current data emphasize the immunomodulating role of vitamin D in enhancing the anti-inflammatory response. Vitamin D deficiency is an established risk factor for developing multiple sclerosis—the autoimmune demyelinating and degenerative disease of the central nervous system. Several studies confirmed that higher vitamin D serum level is associated with better clinical and radiological outcomes in patients with multiple sclerosis, whereas vitamin D supplementation benefits in multiple sclerosis remain inconclusive. Despite that, many experts suggest regular measurements of vitamin D serum levels and supplementation in patients with multiple sclerosis. In this study, 133 patients with multiple sclerosis (relapsing–remitting subtype) were prospectively observed in a 0-, 12- and 24-month time span in a clinical setting. The study group consisted of 71.4% of patients (95 out of 133) supplementing vitamin D. The associations between vitamin D serum levels, clinical outcomes (disability status expressed by EDSS, number of relapses and time to relapse) and radiological outcomes (new T2-weighted lesions and number of gadolinium-enhanced lesions) were evaluated. There were no statistically significant correlations between clinical outcomes and vitamin D serum levels or supplementations. Fewer new T2-weighted lesions were observed in patients with vitamin D supplementations (*p* = 0.034) in 24 months of observation. Moreover, an optimal or higher level of vitamin D (>30 ng/mL) maintained throughout the entire observation period was associated with a lower number of new T2-weighted lesions in 24 months of observation (*p* = 0.045). These results support vitamin D implementation commencement and amelioration in patients with multiple sclerosis.

## 1. Introduction

Vitamin D is well known for maintaining calcium and phosphorus extracellular homeostasis to preserve appropriate bone matrix mineralization [1]. Currently, vitamin D immunomodulating effects are increasingly recognized [2,3]. The active vitamin D metabolite, calcitriol-1,25-hydroxycholecalciferol (1,25(OH)2D3), acts as the vitamin D receptor (VDR) presented in almost every immune cell [4]. Calcitriol modulates the innate and adaptive immune systems, enhancing the anti-inflammatory response [5]. The associations between vitamin D’s immunomodulating role and pathogenesis or potential therapeutic implications of autoimmune diseases have been also researched [6]. Vitamin D deficiency is one of the risk factors for multiple sclerosis (MS), an autoimmune demyelinating and degenerative disease of the central nervous system (CNS) [7]. The causal role of low serum 25-hydroxyvitamin D (25-(OH)D3) levels in developing MS was also confirmed by genome-wide association studies (GWAS) [8,9]. Moreover, several observational studies indicated vitamin D intake may reduce the risk of MS [10,11].

MS is the leading cause of non-traumatic disability in young adults [12,13]. It is estimated that 2.8 million people live with MS worldwide [14]. The course of MS is largely diversified; in most patients, acute worsening of the neurological function (relapse) is followed by a remission period with complete or incomplete recovery of neurological symptoms. The disability progression is assessed by the Expanded Disability Status Scale [15]. Approximately 85% of MS patients develop relapsing–remitting MS (RRMS), whereas 10–15% of patients present with primary progressive MS (PPMS) [16]. Magnetic resonance imaging (MRI) scans detecting demyelinating lesions play a crucial role in the diagnosis and monitoring of the progress of MS [17]. Despite the significant development of disease-modifying therapy (DMT), MS remains an uncurable disease [18].

1,25(OH)D3 inhibits the development of experimental autoimmune encephalomyelitis (EAE), the animal model of CNS inflammation [19]. Vitamin D implementation among MS patients affects the cytokine levels, reducing the synthesis of pro-inflammatory cytokines and elevating the concentrations of the anti-inflammatory cytokines, for example, transforming growth factor-beta (TGF-β) or interleukin-10 (IL-10) [20,21,22]. Vitamin D also activates the oligodendrocytes’ progenitor cell differentiation into mature oligodendrocytes. This process is essential for myelin production and may have a potential role in remyelination [23,24].

Numerous studies evaluated the association between vitamin D serum levels and clinical and radiological outcomes in MS patients. A meta-analysis by Martinez-Lapiscina et al. comprising thirteen qualified studies with nearly 3500 individuals, disclosed that a reduction in relapse rates and radiological activity in MRI was correlated with a rise in serum 25(OH)D levels [25]. Additionally, a meta-analysis by Moosazadeh et al., including fourteen studies (2817 MS patients), indicated that a decrease in the disability expressed by EDSS was associated with 25(OH)D level increases [26].

However, the beneficial role of vitamin D implementation in MS patients remains unresolved. The Cochrane meta-analysis by Jagannath et al. revealed that vitamin D implementation showed no therapeutic effects on clinical MS outcomes as well as gadolinium-enhanced lesions (GELs) in MRI [27]. Analogous results were obtained by McLaughlin et al. Furthermore, only this meta-analysis assessed the effect on new T2-weighted (T2-w) lesions in MRI [28]. Following meta-analyses evaluated only the effect of vitamin D supplementation on clinical outcomes. Yuan et al. disclosed no benefits of vitamin D supplementation on EDSS and relapses throughout research [29]. Meta-analysis by Hanaei et al. revealed no significant impact of vitamin D supplementation on relapse rate and disability [30]. Doosti-Irani et al. indicated no significant benefits of vitamin D implementations on EDSS [31]. In addition, Zeng et al. provided comparable results on EDSS and annualized relapse rates (ARRs) [32]. Detailed information about cited meta-analyses is presented in Table 1.

Based on this current data, many experts still suggest the need for other clinical trials to provide significant evidence for the therapeutic role of vitamin D, together with the need for vitamin D status measurements and supplementation in patients with MS [33,34,35,36]. This study aimed to evaluate the associations between vitamin D serum levels and the clinical and radiological outcomes of MS in both vitamin D supplementing and non-supplementing patients in a clinical setting.

## 2. Materials and Methods

A total of 133 individuals were recruited to the study between October 2018 and April 2021 in the Department of Neurology, Medical University of Silesia in Katowice, Poland. Informed consent for participation in the study was obtained from every patient. The inclusions criteria were:

1. Diagnosed with MS (RRMS, SPMS and PPMS) according to the 2010 or 2017 revised McDonald criteria;
2. Aged over 18 years;
3. EDSS ≤ 6.5;
4. Stable treatment with DMT in a clinical trial or drug prescription program for at least one year at the baseline (interferon beta, glatiramer acetate, dimethyl fumarate, teriflunomide, natalizumab, fingolimod, ocrelizumab, alemtuzumab and cladribine).

The exclusions criteria were as follow:

1. Relapse in the last 4 weeks;
2. EDSS > 6.5;
3. Pregnancy or breast-feeding;
4. Acute or chronic renal failure;
5. Hypercalcemia in medical history;
6. DMT notable ineffectiveness is defined as at least two new T2-w lesions and one GEL in MRI and two relapses in a year; DMT discontinuation/termination; or DMT severe adverse effect.

One of the exclusion criteria was an ineffectiveness of DMT (defined as at least two new T2-w lesions and one GEL in MRI and two relapses in a year) that required implementation of highly effective treatment to reduce the impact of treatment modifications on outcomes in MS patients. Moreover, most of the participants were enrolled to study between October and May to reduce to impact of insolation on vitamin D serum levels. Patients who were enrolled between June and September declared not effective sun exposure (sunbathing at least 15 min/day between 10 am and 3 pm, exposing forearms and lower legs and not wearing sunscreen) [37].

At the baseline, recruited patients fulfilled a “Vitamin D supplementation and factors influencing vitamin D status questionnaire”. The supporting material is available in Supplementary Material. Vitamin D serum levels were measured at every time point. According to the Polish endocrine experts’ recommendations, the following ranges of vitamin D status were applied: severe deficiency (0–10 ng/mL), deficiency (>10–20 ng/mL), suboptimal (>20–30 ng/mL), optimal (>30–50 ng/mL), high (>50–100 ng/mL) and toxic (>100 ng/mL) [38]. The study protocol did not institute any intervention. The associations between vitamin D serum levels and the following clinical and radiological outcomes were evaluated at every time point:

1. EDSS change;
2. Number of new relapses;
3. Time to relapse;
4. Number of new or enlarged T2-w lesions in MRI;
5. Number of GELs in MRI.

The statistical analysis was performed using Statistica 13.3 (TIBCO Software Inc. (2017) Statistica (data analysis software system, version 13., http://statistica.io)). The quantitative variables are presented as the arithmetic mean and standard deviation (normally distributed variables) or the median and interquartile range (variables of abnormal/skewed distribution). The normality of distribution was assessed using the Shapiro–Wilk test. Qualitative variables are presented as absolute values and percentages.

As the normal distribution in the analyzed groups was not confirmed, the intergroup differences for the quantitative variable were assessed using the Mann–Whitney U test or the Kruskal–Wallis test (variables of skewed distribution). In the case of statistically significant differences within many groups revealed by the Kruskal–Wallis test, a post hoc type analysis was performed. Fisher’s exact test or chi-square test was performed for the qualitative variable.

## 3. Results

In total, 133 patients were recruited in this study. The study group included 37 men and 96 women. The median age was 40.47 (±10.24) years, and the mean disease duration was 10.68 (±6.71) years at the baseline. All patients were diagnosed with RRMS. The characteristics of the studied group are shown in Table 2.

**Figure 1:**
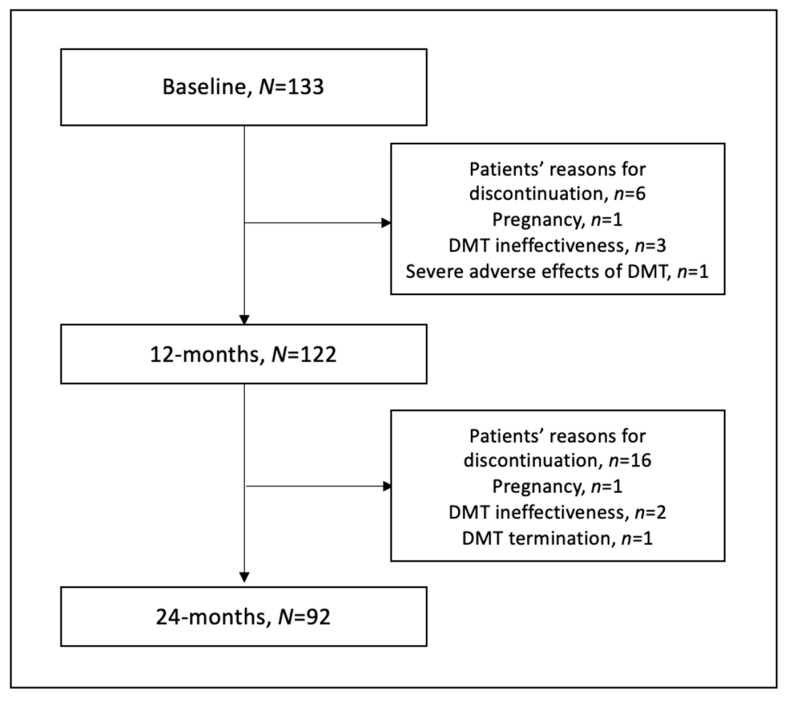
Flowchart of the study.

The 122 patients were assessed at 12 months and 92 patients at 24 months of observation. The main cause of study discontinuation was the patient’s reasons (resignation or failing to control visits) but also the ineffectiveness of DMT, DMT discontinuation or termination, DMT severe adverse effects and pregnancy (Figure 1). There were two cases of pregnancy; the first woman was administered with dimethyl fumarate and the second with fingolimod. Both cases required DMT modification. There was also one case of a severe adverse effect of DMT, prolonged lymphopenia during dimethyl fumarate treatment of grade 3, with absolute lymphocytes count (ALC) ranging 200–499/μL, resulting in drug discontinuation.

### 3.1. Vitamin D Status and Supplementation

At the baseline, the mean vitamin D serum level was 35.77 ng/mL in the study group. In total, 71.4% (*n* = 95) of participants proclaimed the vitamin D implementation. The administered doses ranged from 1000 to 4000 IU daily. All patients were administered cholecalciferol, which was taken orally. There were no cases of gastrointestinal disorders or malabsorption in the participants’ medical histories. The mean vitamin D serum level was 42.03 ng/mL among participants with supplementation and 20.12 ng/mL among patients without supplementation. The detailed vitamin D serum levels and vitamin D status in subgroups are presented in Table 3.

### 3.2. Vitamin D and Clinical Outcomes

In the study, no statistically significant correlations were found between vitamin D serum levels and the patient disability expressed by EDSS among patients in the study group at the baseline, 12 months, and 24 months of observations in both groups (with and without supplementations). Similarly, the number of new relapses, as well as the time to relapse, did not correspond with vitamin D serum levels in the study group. There were no statistically significant differences between patients with and without vitamin D supplementation (Table 4).

### 3.3. Vitamin D and Radiological Outcomes

Patients with vitamin D supplementation had statistically fewer new T2-w lesions (*p* = 0.034) but no GELs (*p* = 0.094) in 24 months of observation (Table 3). Moreover, an optimal or higher level of vitamin D (>30 ng/mL) maintained throughout the entire observation period was associated with a lower number of new cumulative T2-w lesions in MRI in 24 months of observations (*p* = 0.045). There were no statistically significant differences between patients who had at least once measured insufficient levels of vitamin D. This positive association was statistically significant in the second year of observation (*p* = 0.044). Additionally, patients with optimal or higher levels had a reduced number of GELs but only at 12 months of observation (*p* = 0.006). The results are presented in Table 5.

### 3.4. Vitamin D and Disease-Modifying Therapy

Patients with vitamin D supplementation treated with interferon beta, glatiramer acetate, dimethyl fumarate and teriflunomide had statistically fewer new T2-w lesions (*p* = 0.040). For patients treated with more effective drugs (fingolimod, natalizumab, ocrelizumab and alemtuzumab), the radiological benefits of supplementation were not confirmed. The results are presented in Table 6.

## 4. Discussion

Firstly, the study reports vitamin D status and supplementation among patients with MS in clinical settings. Secondly, the study provides data about the associations between vitamin D serum levels and the clinical and radiological outcomes of MS patients. The study concerned RRMS patients both with vitamin D supplementation and without supplementation. However, numerous studies confirmed that an increase in vitamin D serum levels is related to limiting the clinical and radiological indicators of disease progression, but the therapeutic role of vitamin D implementation in MS patients remains unsettled.

### 4.1. Vitamin D Status and Supplementation

At the baseline, insufficient vitamin D serum levels (equal to or lower than 30 ng/mL) were reported in 61 out of 133 patients (45.9%), 31 out of 92 (79%) in patients with supplementations and 30 out of 38 (%) in patients without supplementation. After 24 months of observations, insufficient vitamin D serum levels were reported in 27,42% of patients with supplementation and 70% of patients without supplementation. What is more, at the baseline, 21.1% of patients without supplementations had a severe deficiency of vitamin D. The Scandinavian experts recommend systematic vitamin D serum level evaluation in MS patients and supplementation to reach the optimal level (30–50 ng/mL) [39]. Moreover, other experts suggest supplementation to achieve 40 ng/mL, especially in young patients [40].

Furthermore, 95 out of 133 patients declared vitamin D supplementation (71.4%) in the study group. This percentage is comparable with another study in the Polish population, in which 103 out of 139 patients were supplementing vitamin D [41]. A lower percentage of MS patients supplementing vitamin D was reported by Pape et al. (60.0%) [42] and Masullo et al. (52.78%) [43]. The vitamin D status in MS patients is still insufficient, and a significant number of patients do not supplement vitamin D.

### 4.2. Vitamin D and Clinical Outcomes

In this study, vitamin D status was not associated with the level of MS patients’ disability expressed by EDSS. In addition, other authors have provided similar results [44,45]. However, Brola et al. indicated a statistically significant connection between vitamin D serum levels and relapse instances, as well as the level of disability in MS patients [46]. In addition, a vitamin D serum level higher than 20 ng/mL was associated with a 2.78 times greater probability of a lower level of disability (EDSS < 4.0) in MS patients [47]. Similar correlations were confirmed in other studies [48,49]. Furthermore, there were no statistically significant associations between vitamin D serum levels and the number of new relapses in this study. Feree’ et al. provided similar results among fingolimod-treated patients with MS. However, a correlation between low vitamin D serum levels and a higher relapse rate was observed in natalizumab-treated patients with MS [50]. In addition, other authors revealed correlations between low vitamin D serum levels and higher relapse risk in patients with MS [51,52,53].

Moreover, there were no statistically significant differences between patients with vitamin D supplementation and without supplementation in this study according to the clinical outcomes in MS, whereas Burton et al. revealed that vitamin D supplementation benefits lower growth in EDSS and reduces mean ARR [54]. However, further research did not confirm vitamin D supplementation’s impact on EDSS and relapse rates in patients with MS [55,56,57].

Similar to the majority of authors, this study did not reveal any benefits of vitamin D supplementation or proper vitamin D status on the clinical outcomes of MS.

### 4.3. Vitamin D and Radiological Outcomes

Statistically significant associations between vitamin D serum levels and a reduced number of new T2-w lesions as well as GELs in MRI in MS patients were reported in this study. Five years of observation of patients with CIS (BENEFIT study) showed that an increment in average vitamin D serum levels (20 ng/mL) was correlated with a half lower rate of new GELs and a quarter lower yearly increase in T2-w lesion volume and lower yearly brain atrophy [58]. What is more, a 20 ng/mL increase in vitamin D serum levels resulted in lowering GELs in patients with MS in the first two years of treatment with INF-β1b [59]. Similar results were also observed in the CILMB study [60]. Furthermore, an increase in vitamin D serum levels was associated with a 15% lower risk of new T2-w demyelinating lesions and a 32% lower risk of GELS in MRI [61]. This study supports the protective impact of optimal or higher vitamin D status levels on radiological outcomes.

Additionally, in this study, vitamin D supplementation was associated with a reduced number of new T2-w lesions in MRI in patients with MS at 2 years of observation. Similar correlations were observed by several RCTs concerning the benefits of vitamin D supplementations in MS patients [62,63,64], whereas other RCTs did not provide statistically significant results [65,66,67]. The characteristic features of RCTs assessing the impact of vitamin D supplementation on radiological outcomes in MS patients are presented in Table 7.

### 4.4. Vitamin D and Disease-Modifying Therapy

The radiological benefits of vitamin D supplementations were observed in patients treated with interferon beta, glatiramer acetate, dimethyl fumarate and teriflunomide but not with more effective drugs, such as fingolimod, natalizumab, ocrelizumab and alemtuzumab. This is probably due to the relatively small group size and higher radiological effectiveness of these treatments.

### 4.5. Study Strengths and Limitations

This study provides data from 24 months of observation of vitamin D serum levels and clinical and radiological outcomes of patients with MS in a clinical setting. Only RRMS patients were recruited due to the necessity of annual visits with MRI assessment. Consequently, we included patients with almost all types of DMT. The inclusion and exclusion criteria were strict regarding DMT treatment (stable treatment for at least a year and elimination from the study due to any DMT ineffectiveness or discontinuation) to evaluate the associations between vitamin D serum levels and clinical and radiological outcomes. What is more, the vitamin D serum level was measured at every time point (baseline, 12 months and 24 months), whereas in previous observational studies, the clinical and radiological outcomes were correlated only to basal vitamin D serum levels. This study did not evaluate calcium, phosphorus and the parathyroid hormone in determining the biological effect of vitamin D in patients with multiple sclerosis. In our opinion, this requires further studies. At the baseline, the study group was divided into two subgroups, with and without supplementations, to assess the differences between them. The supplementation of vitamin D in patients was recommended according to the Polish Endocrine Association’s experts, where the study methods did not assume the placebo treatment against vitamin D implementation in patients with multiple sclerosis.

## 5. Conclusions

The associations between vitamin D serum levels and radiological but no clinical outcomes of patients with MS were reported in this study. There were no statistically significant correlations between vitamin D serum levels and the level of disability expressed by EDSS or the number of new relapses, whereas a higher vitamin D serum level was associated with a reduced number of new T2-w lesions as well as GELs in MRI in patients with MS independent of vitamin D supplementation. Furthermore, vitamin D supplementation was related to a reduced number of new T2-w lesions in MRI among MS patients at 2 years of observation. Sufficient vitamin D serum levels, along with supplementation, may have some benefits in MS patients. Although 71.4% of participants declared vitamin D implementation, the vitamin D status among patients with multiple sclerosis is still insufficient. Supplementation commencement and enhancements should be put into practice in MS patients.

## Figures and Tables

**Table 1:** Meta-analyses assessing the impact of vitamin D supplementation on clinical and radiological outcomes among patients with multiple sclerosis.

Author, Year [Ref.]	Number of Eligible RCTs	Study Group/Control Group	Clinical and Radiological Outcomes			
			Relapses/ ARRs	EDSS	GELs	New or Enlarged T2 Lesions
Jagannath, 2018 [27]	12	464/469	NS	NS	NS	-
McLaughlin, 2018 [28]	12	479/468	NS	NS	NS	NS
Yuan, 2021 [29]	9	356/362	NS	NS	-	-
Hanaei, 2021 [30]	9	245/247	NS	NS	-	-
Doosti-Irani, 2019 [31]	6	166/165	-	NS	-	-
Zheng, 2018 [32]	6	169/168	NS	NS	-	-

Note: RCTs—randomized controlled trials; ARR—annualized relapse rate; EDSS—Expanded Disability Status Scale; GELs—gadolinium-enhanced lesions; T2—T2-weighted lesions; NS—non-significant; -—not tested.

**Table 2:** Demographic and clinical characteristics of the studied group at the baseline, 12 months and 24 months.

Characteristics	Baseline (*N* = 133)	12 Months (*N =* 120)	24 Months (*N* = 90)
Men/Women (*n*, %)	37 (27.8%)/96 (72.2%)	35 (29.2%)/85 (70.8%)	29 (32.2%)/61 (77.8%)
EDSS (median, min–max)	2.5 (0–6.5)	2.5 (0–6.5)	2.5 (1–6.5)
Vitamin D serum level (mean ± SD)	35.77 ± 25.23 ng/mL	37.96 ± 24.20 ng/mL	37.42 ± 21.293 ng/mL
*Patients with supplementation (mean)*	*42.03 ng/mL*	*43.32 ng/mL*	*42.31 ng/mL*
*Patients without supplementation (mean)*	*20.12 ng/mL*	*25.71 ng/mL*	*27.91 ng/mL*
DMT (*n*, %)			
Interferon-beta	22 (16.5%)	21 (15.8%)	11 (8.3%)
Glatiramer acetate	10 (7.5%)	7 (5.3%)	7 (5.3%)
Dimethyl fumarate	65 (48.9%)	58 (43.6%)	44 (33.1%)
Teriflunomide	15 (11.3%)	14 (10.5%)	14 (10.5%)
Fingolimod	11 (8.3%)	9 (6.8%)	8 (6.0%)
Natalizumab	8 (6.0%)	9 (6.8%)	8 (6.0%)
Ocrelizumab	1 (0.8%)	1 (0.8%)	1 (0.8%)
Alemtuzumab	1 (0.8%)	1 (0.8%)	0 (0.0%)

Note: EDSS—Expanded Disability Status Scale; SD—standard deviation; DMT—disease-modifying therapy.

**Table 3:** Mean vitamin D serum levels and vitamin D status among patients with and without supplementation in the study group.

	Patients with Supplementation			Patients without Supplementation		
	Baseline (*n* = 95)	12 Months (*n* = 86)	24 Months (*n* = 62)	Baseline (*n* = 38)	12 Months (*n* = 36)	24 Months (*n* = 30)
Vitamin D serum level (mean ± SD) (ng/mL)	42.58 ± 26.89	43.81 ± 25.58	42.31 ± 21.80	20.58 ± 11.88	25.45 ± 15.04	27.40 ± 16.03
Severe deficiency (*n*, %)	1 (1.05%)	0 (0%)	1 (1.61%)	8 (21.1%)	5 (13.9%)	3 (10.0%)
Deficiency (*n*, %)	10 (10.53%)	7 (7.37%)	7 (11.29%)	14 (36.8%)	12 (33.3%)	8 (26.7%)
Suboptimal (*n*, %)	20 (21.05%)	19 (20.0%)	9 (14.52%)	8 (21.1%)	9 (25%)	10 (33.3%)
Optimal (*n*, %)	41 (43.16%)	38 (40.0%)	28 (45.16%)	7 (18.4%)	6 (11.1%)	6 (10.0%)
High (*n*, %)	19 (20%)	20 (21.05%)	16 (25.81)	1 (2.6%)	4 (11.1)	3 (10.0%)
Toxic (*n*, %)	4 (4.21%)	2 (2.11%)	1 (1.6%)	0 (0%)	0 (0%)	0 (0%)

Note: severe deficiency (0–10 ng/mL); deficiency (>10–20 ng/mL); suboptimal (>20–30 ng/mL); optimal (>30–50 ng/mL); high (>50–100 ng/mL); toxic (>100 ng/mL).

**Table 4:** Clinical and radiological outcomes in patients with and without vitamin D supplementation at 24 months of observations.

	Patients without Supplementation		Patients with Supplementation		
24 Months of Observation	Mean ± SD	Median (IQR)	Mean ± SD	Median (IQR)	*p*
EDSS	2.9 (±1.6)	2.5 (1.5–4.5)	2.7 (±1.4)	2.5 (1.5–3.5)	0.61548
New relapses	0.2 (±0.5)	0.0 (0.0–0.0)	0.1(±0.4)	0.0 (0.0–0.0)	0.69798
Number of new T2	0.8 (+0.4)	0.0 (0.0–1.0)	0.4 (±1.1)	0.0 (0.0–0.0)	0.03443
Number of GELs	0.8 (+0.0)	0.0 (0.0–1.0)	0.0 (±11)	0.0 (0.0–1.0)	0.09407

Note: SD—standard deviation; IQR—interquartile range; EDSS—Expanded Disability Status Scale; T2—T2-weighted lesion in magnetic resonance imaging; GELs—gadolinium-enhanced lesions.

**Table 5:** Radiological outcomes in patients with periods with optimal or higher vitamin D serum levels (>30 ng/mL) at baseline, 12 months and 24 months of observation.

	Number of 12-Month Periods with Optimal or Higher Vitamin D Serum Level (>30 ng/mL)				
	0	1	2	3	
Radiological Outcomes	Mean ± SD	Mean ± SD	Mean ± SD	Mean ± SD	*p*
**Number of new T2**					
Baseline	0.3 (±0.6)	0.3 (±0.4)	0.4 (±1.2)	0.2 (±0.4)	0.20610
12 months	0.3 (±0.7)	0.3 (±0.6)	0.2 (±0.5)	0.1 (±0.2)	0.28050
24 months	0.4 (±1.1)	0.4 (±0.6)	0.6 (±1.2)	0.1 (±0.2)	0.04440
Cumulative	1.1 (±1.2)	1.0 (±0.6)	1.2 (±1.9)	0.4 (±0.4)	0.04570
**Number of GELs**					
Baseline	0.2 (±0.6)	0.5 (±1.0)	0.3 (±1.1)	0.1 (±0.3)	0.00620
12 months	0.3 (±0.8)	0.2 (±0.8)	0.2 (±0.8)	0.1 (±0.3)	0.54180
24 months	0.1 (±0.6)	0.3 (±0.6)	0.0 (±0.0)	0.2 (±0.5)	0.45940
Cumulative	0.5 (±1.3)	0.8 (±1.5)	0.6 (±1.4)	0.3 (±0.9)	0.12770

Note: SD—standard deviation; T2–T2-weighted lesion in magnetic resonance imaging; GELs–gadolinium-enhanced lesions.

**Table 6:** The effects of vitamin D supplementation on clinical and radiological outcomes in multiple sclerosis depending on the disease-modifying therapy.

		Patients without Supplementation		Patients with Supplementation		
	24 Months of Observation	Mean ± SD	Median (IQR)	Mean ± SD	Median (IQR)	*p*
Interferon beta glatiramer acetate, dimethyl fumarate, teriflunomide.	New relapses	0.7 (±1.3)	0.0 (0.0–1.0)	0.6(±1.3)	0.0 (0.0–2.0)	0.58962
	Number of new T2	0.9 (±1.6)	0.0 (0.0–1.0)	0.4 (±1.0)	0.0 (0.0–2.0)	0.04041
	Number of GELs	0.8 (±1.7)	0.0 (0.0–1.0)	0.4 (±11)	0.0 (0.0–1.0)	0.07645
Fingolimod, natalizumab, ocrelizumab, alemtuzumab.	New relapses	0.8 (±1.2)	0.0 (0.0–0.0)	1.2 (±1.2)	0.0 (0.0–0.0)	0.78988
	Number of new T2	0.5 (±0.8)	0.0 (0.0–1.0)	0.5 (±1.3)	0.0 (0.0–0.0)	0.73330
	Number of GELs	0.3 (±0.5)	0.0 (0.0–1.0)	0.7 (±1.6)	0.0 (0.0–1.0)	0.20511

Note: SD—standard deviation; IQR—interquartile range; EDSS—Expanded Disability Status Scale; T2—T2-weighted lesion in magnetic resonance imaging; GELs—gadolinium-enhanced lesions.

**Table 7:** Randomized clinical trials evaluating the impact of vitamin D supplementation on radiological outcomes among patients with multiple sclerosis.

Author, Year [Ref.]	Study Group/Control Group	Vitamin D Doses		Vitamin D Form	Time of Supplementation [Months]	Radiological Outcomes in Patients with MS
		Study Group	Control Group			
Camu, 2019 [62]	45/45	100,000 IU/5 days	Placebo	D3	24	Reduction in new T1 lesions (*p* = 0.025); reduction in T1 lesion volume (*p* = 0.031)
Derakhshand, 2013 [65]	15/15	50,000 IU/week	Placebo	D3	12	NS
Golan, 2013 [57]	24/21	4370 IU/day	800 IU/day	D3	12	NS
Hupperts, 2019 [63]	113/116	14,007 IU/day	Placebo	D3	12	Reduction of GELs (*p* = 0.0045); reduction in increase of total T2 lesion volume (*p* = 0.035)
Mosayebi, 2011 [21]	26/33	300,000 IU/month (i.m.)	Placebo	D3	6	NS
O’Connell *, 2017 [66]	23/9	5000 IU/day or 10,000 IU/day	Placebo	D3	6	NS
Soilu-Hanninen, 2012 [64]	34/32	20,000 IU/week	Placebo	D3	12	Reduction of GELs (*p* = 0.004)
Stein, 2011 [67]	11/12	6000–12,000 IU/day + 1000 IU/day	Placebo	D2	6	NS

Note: * Study among patients with the clinically isolated syndrome (CIS); D3—cholecalciferol; D2—ergocalciferol; T1—T1-weighted lesions in magnetic resonance imaging; T2—T2-weighted lesions in magnetic resonance imaging, GELs—gadolinium-enhanced lesions; NS—non-significant.

## Data Availability

The data presented in this study are not publicly available due to privacy restrictions.

## Supplementary Materials

The following supporting information can be downloaded at: https://www.mdpi.com/article/10.3390/nu15061465/s1, Vitamin D supplementation and factors influencing vitamin D status questionnaire.

## References

[1] Gil Á., Plaza-Diaz J., Mesa M.D. (2018). Vitamin D: Classic and Novel Actions. Ann. Nutr. Metab..

[2] Bivona G., Agnello L., Ciaccio M. (2018). The immunological implication of the new vitamin D metabolism. Cent. Eur. J. Immunol..

[3] Sassi F., Tamone C., D’Amelio P. (2018). Vitamin D: Nutrient, Hormone, and Immunomodulator. Nutrients.

[4] Provvedini D.M., Tsoukas C.D., Deftos L.J., Manolagas S.C. (1983). 1,25-dihydroxyvitamin D3 receptors in human leukocytes. Science.

[5] Prietl B., Treiber G., Pieber T.R., Amrein K. (2013). Vitamin D and immune function. Nutrients.

[6] Bellan M., Andreoli L., Mele C., Sainaghi P.P., Rigamonti C., Piantoni S., De Benedittis C., Aimaretti G., Pirisi M., Marzullo P. (2020). Pathophysiological Role and Therapeutic Implications of Vitamin D in Autoimmunity: Focus on Chronic Autoimmune Diseases. Nutrients.

[7] Ascherio A., Munger K. (2016). Epidemiology of Multiple Sclerosis: From Risk Factors to Prevention—An Update. Semin. Neurol..

[8] Rhead B., Bäärnhielm M., Gianfrancesco M., Mok A., Shao X., Quach H., Shen L., Schaefer C., Link J., Gyllenberg A. (2016). Mendelian randomization shows a causal effect of low vitamin D on multiple sclerosis risk. Neurol. Genet..

[9] Wang R. (2022). Mendelian randomization study updates the effect of 25-hydroxyvitamin D levels on the risk of multiple sclerosis. J. Transl. Med..

[10] Munger K.L., Levin L.I., Hollis B.W., Howard N.S., Ascherio A. (2006). Serum 25-hydroxyvitamin D levels and risk of multiple sclerosis. JAMA.

[11] Munger K.L., Zhang S.M., O’Reilly E., Hernán M.A., Olek M.J., Willett W.C., Ascherio A. (2004). Vitamin D intake and incidence of multiple sclerosis. Neurology.

[12] Compston A., Coles A. (2008). Multiple sclerosis. Lancet.

[13] Browne P., Chandraratna D., Angood C., Tremlett H., Baker C., Taylor B.V., Thompson A.J. (2014). Atlas of multiple sclerosis 2013: A growing global problem with widespread inequity. Neurology.

[14] Walton C., King R., Rechtman L., Kaye W., Leray E., Marrie R.A., Robertson N., La Rocca N., Uitdehaag B., van der Mei I. (2020). Rising prevalence of multiple sclerosis worldwide: Insights from the Atlas of MS, third edition. Mult. Scler. J..

[15] Kurtzke J.F. (1983). Rating neurologic impairment in multiple sclerosis: An expanded disability status scale (EDSS). Neurology.

[16] Lublin F.D., Coetzee T., Cohen J.A., Marrie R.A., Thompson A.J., International Advisory Committee on Clinical Trials in MS (2020). The 2013 clinical course descriptors for multiple sclerosis. A clarification. Neurology.

[17] Hemond C.C., Bakshi R. (2018). Magnetic Resonance Imaging in Multiple Sclerosis. Cold Spring Harb. Perspect. Med..

[18] Hauser S.L., Cree B.A.C. (2020). Treatment of Multiple Sclerosis: A Review. Am. J. Med..

[19] Pedersen L.B., Nashold F.E., Spach K.M., Hayes C.E. (2007). 1,25-hydroxyvitamin D3 reverses experimental autoimmune encephalomyelitis by inhibiting chemokine synthesis and monocyte trafficking. J. Neurosci. Res..

[20] Sotirchos E.S., Bhargava P., Eckstein C., Van Haren K., Baynes M., Ntranos A., Gocke A., Steinman L., Mowry E.M., Calabresi P.A. (2016). Safety and Immunologic Effects of High- vs Low-Dose Cholecalciferol in Multiple Sclerosis. Neurology.

[21] Mosayebi G., Ghazavi A., Ghasami K., Jand Y., Kokhaei P. (2011). Therapeutic effect of vitamin D3 in multiple sclerosis patients. Immunol. Investig..

[22] Walawska-Hrycek A., Galus W., Hrycek E., Kaczmarczyk A., Krzystanek E. (2021). The Impact of Vitamin D Low Doses on Its Serum Level and Cytokine Profile in Multiple Sclerosis Patients. J. Clin. Med..

[23] de la Fuente A.G., Errea O., van Wijngaarden P., Gonzalez G.A., Kerninon C., Jarjour A.A., Lewis H.J., Jones C.A., Nait-Oumesmar B., Zhao C. (2015). Vitamin D receptor-retinoid X receptor heterodimer signaling regulates oligodendrocyte progenitor cell differentiation. J. Cell Biol..

[24] Gomez-Pinedo U., Cuevas J.A., Benito-Martín M.S., Moreno-Jiménez L., Esteban-Garcia N., Torre-Fuentes L., Matías-Guiu J.A., Pytel V., Montero P., Matías-Guiu J. (2020). Vitamin D increases remyelination by promoting oligodendrocyte lineage differentiation. Brain Behav..

[25] Martínez-Lapiscina E.H., Mahatanan R., Lee C.-H., Charoenpong P., Hong J.-P. (2020). Associations of serum 25(OH) vitamin D levels with clinical and radiological outcomes in multiple sclerosis, a systematic review and meta-analysis. J. Neurol. Sci..

[26] Moosazadeh M., Nabinezhad-Male F., Afshari M., Nasehi M.M., Shabani M., Kheradmand M., Aghaei I. (2021). Vitamin D status and disability among patients with multiple sclerosis: A systematic review and meta-analysis. AIMS Neurosci..

[27] Jagannath V.A., Filippini G., Di Pietrantonj C., Asokan G.V., Robak E.W., Whamond L., Robinson S.A. (2018). Vitamin D for the management of multiple sclerosis. Cochrane Database Syst. Rev..

[28] McLaughlin L., Clarke L., Khalilidehkordi E., Butzkueven H., Taylor B., Broadley S.A. (2018). Vitamin D for the treatment of multiple sclerosis: A meta-analysis. J. Neurol..

[29] Yuan X., Guo L., Jiang C., Yang X., Huang J. (2021). The Effect of Different Administration Time and Dosage of Vitamin D Supplementation in Patients with Multiple Sclerosis: A Meta-Analysis of Randomized Controlled Trials. Neuroimmunomodulation.

[30] Hanaei S., Ali Sahraian M., Mohammadifar M., Ramagopalan S.V., Ghajarzadeh M. (2021). Effect of Vitamin D Supplements on Relapse Rate and Expanded Disability Status Scale (EDSS) in Multiple Sclerosis (MS): A Systematic Review and Meta-Analysis. Int. J. Prev. Med..

[31] Doosti-Irani A., Tamtaji O.R., Mansournia M.A., Mobarhan M.G., Ferns G., Kakhaki R.D., Shahmirzadi A.R., Asemi Z. (2019). The effects of vitamin D supplementation on expanded disability status scale in people with multiple sclerosis: A critical, systematic review and meta-analysis of randomized controlled trials. Clin. Neurol. Neurosurg..

[32] Zheng C., He L., Liu L., Zhu J., Jin T. (2018). The efficacy of vitamin D in multiple sclerosis: A meta-analysis. Mult. Scler. Relat. Disord..

[33] Fatima M., Lamis A., Siddiqui S.W., Ashok T., Patni N., Fadiora O.E. (2022). Therapeutic Role of Vitamin D in Multiple Sclerosis: An Essentially Contested Concept. Cureus.

[34] Gandhi F., Jhaveri S., Avanthika C., Singh A., Jain N., Gulraiz A., Shah P., Nasir F. (2021). Impact of Vitamin D Supplementation in Multiple Sclerosis. Cureus.

[35] Sintzel M.B., Rametta M., Reder A.T. (2018). Vitamin D and Multiple Sclerosis: A Comprehensive Review. Neurol. Ther..

[36] Pierrot-Deseilligny C., Souberbielle J.-C. (2017). Vitamin D and multiple sclerosis: An update. Mult. Scler. Relat. Disord..

[37] Rusińska A., Płudowski P., Walczak M., Borszewska-Kornacka M.K., Bossowski A., Chlebna-Sokół D., Czech-Kowalska J., Dobrzańska A., Franek E., Helwich E. (2018). Vitamin D Supplementation Guidelines for General Population and Groups at Risk of Vitamin D Deficiency in Poland—Recommendations of the Polish Society of Pediatric Endocrinology and Diabetes and the Expert Panel with Participation of National Specialist Consultant and Representatives of Scientific Societies—2018 Update. Front. Endocrinol. Lausanne.

[38] Płudowski P., Karczmarewicz E., Bayer M., Carter G., Chlebna-Sokół D., Czech-Kowalska J., Dębski R., Decsi T., Do-brzańska A., Franek E. (2013). Practical guidelines for the supplementation of vitamin D and the treatment of deficits in Central Europe—Recommended vitamin D intakes in the general population and groups at risk of vitamin D deficiency. Endokrynol. Pol..

[39] Holmøy T., Torkildsen Ø., Myhr K.-M., Løken-Amsrud K.I. (2012). Vitamin D supplementation and monitoring in multiple sclerosis: Who, when and wherefore. Acta Neurol. Scand. Suppl..

[40] Boltjes R., Knippenberg S., Gerlach O., Hupperts R., Damoiseaux J. (2021). Vitamin D supplementation in multiple sclerosis: An expert opinion based on the review of current evidence. Expert Rev. Neurother..

[41] Galus W., Walawska-Hrycek A., Rzepka M., Krzystanek E. (2022). Vitamin D Supplementation Practices among Multiple Sclerosis Patients and Professionals. J. Clin. Med..

[42] Pape K., Steffen F., Zipp F., Bittner S. (2020). Supplementary medication in multiple sclerosis: Real-world experience and potential interference with neurofilament light chain measurement. Mult. Scler. J. Exp. Transl. Clin..

[43] Masulo L., Papas M.A., Cotugna N., Baker S., Mahoney L., Trabulsi J. (2015). Complemtary and alternative medicine use and nutrient intake among individuals with multiple sclerosis in the United States. J. Community Health.

[44] Fragoso Y.D., Adoni T., Alves-Leon S.V., Apostolos-Pereira S.L., Arruda W.O., Brooks J.B., Cal H.S., Damasceno C.A., Gama P.D., Goncalves M.V. (2017). No correlation was observed between vitamin D levels and disability of patients with multiple sclerosis between latitudes 18° and 30° South. Arq. Neuropsiquiatr..

[45] Rito Y., Flores J., Fernández-Aguilar A., Escalante-Membrillo C., Barboza M.A., Amezcua L., Corona T. (2018). Vitamin D and disability in relapsing—Remitting multiple sclerosis in patients with a Mexican background. Acta Neurol. Belg..

[46] Brola W., Sobolewski P., Szczuchniak W., Góral A., Fudala M., Przybylski W., Opara J. (2016). Association of seasonal serum 25-hydroxyvitamin D levels with disability and relapses in relapsing-remitting multiple sclerosis. Eur. J. Clin. Nutr..

[47] Thouvenot E., Orsini M., Daures J.-P., Camu W. (2015). Vitamin D is associated with degree of disability in patients with fully ambulatory relapsing-remitting multiple sclerosis. Eur. J. Neurol..

[48] Harandi A.A., Shahbeigi S., Pakdaman H., Fereshtehnejad S.-M., Nikravesh E., Jalilzadeh R. (2012). Association of serum 25(OH) vitamin D3 concentration with severity of multiple sclerosis. Iran. J. Neurol..

[49] van der Mei I.A.F., Ponsonby A.-L., Dwyer T., Blizzard L., Taylor B.V., Kilpatrick T., Butzkueven H., McMichael A.J. (2007). Vitamin D levels in people with multiple sclerosis and community controls in Tasmania, Australia. J. Neurol..

[50] Scott T.F., Hackett C.T., Dworek D.C., Schramke C.J. (2013). Low vitamin D level is associated with higher relapse rate in natalizumab treated MS patients. J. Neurol. Sci..

[51] Wang C., Zeng Z., Wang B., Guo S. (2018). Lower 25-hydroxyvitamin D is associated with higher relapse risk in patients with relapsing-remitting multiple sclerosis. J. Nutr. Health Aging.

[52] Runia T.F., Hop W.C.J., de Rijke Y.B., Buljevac D., Hintzen R.Q. (2012). Lower serum vitamin D levels are associated with a higher relapse risk in multiple sclerosis. Neurology.

[53] Simpson S., Taylor B., Blizzard L., Ponsonby A.L., Pittas F., Tremlett H., Dwyer T., Gies P., van der Mei I. (2010). Higher 25-hydroxyvitamin D is associated with lower relapse risk in multiple sclerosis. Ann. Neurol..

[54] Burton J.M., Kimball S., Vieth R., Bar-Or A., Dosch H.M., Cheung R., Gagne D., D’Souza C., Ursell M., O’Connor P. (2010). A phase I/II dose-escalation trial of vitamin D3 and calcium in multiple sclerosis. Neurology.

[55] Shaygannejad V., Janghorbani M., Ashtari F., Dehghan H. (2012). Effects of adjunct low-dose vitamin d on relapsing-remitting multiple sclerosis progression: Preliminary findings of a randomized placebo-controlled trial. Mult. Scler. Int..

[56] Kampman M.T., Steffensen L.H., Mellgren S.I., Jørgensen L. (2012). Effect of vitamin D3 supplementation on relapses, disease progression, and measures of function in persons with multiple sclerosis: Exploratory outcomes from a double-blind randomised controlled trial. Mult. Scler..

[57] Golan D., Halhal B., Glass-Marmor L., Staun-Ram E., Rozenberg O., Lavi I., Dishon S., Barak M., Ish-Shalom S., Miller A. (2013). Vitamin D supplementation for patients with multiple sclerosis treated with interferon-beta: A randomized controlled trial assessing the effect on flu-like symptoms and immunomodulatory properties. BMC Neurol..

[58] Ascherio A., Munger K.L., White R., Köchert K., Simon K.C., Polman C.H., Freedman M.S., Hartung H.P., Miller D.H., Montalbán X. (2014). Vitamin D as an early predictor of multiple sclerosis activity and progression. JAMA Neurol..

[59] Simpson S.J., van der Mei I., Lucas R.M., Ponsonby A.-L., Broadley S., Blizzard L., Taylor B., Dear K., Dwyer T., Ausimmune/AusLong Investigators Group (2018). Sun exposure across the life course significantly modulates early multiple sclerosis clinical course. Front. Neurol..

[60] Rotstein D.L., Healy B.C., Malik M.T., Carruthers R.L., Musallam A.J., Kivisakk P., Weiner H.L., Glanz B., Chitnis T. (2015). Effect of vitamin D on MS activity by disease-modifying therapy class. Neurol. Neuroimmunol. Neuroinflamm..

[61] Mowry E.M., Waubant E., McCulloch C.E., Okuda D.T., Evangelista A.A., Lincoln R.R., Gourraud P.A., Brenneman D., Owen M.C., Qualley P. (2012). Vitamin D status predicts new brain magnetic resonance imaging activity in multiple sclerosis. Ann. Neurol..

[62] Camu W., Lehert P., Pierrot-Deseilligny C., Hautecoeur P., Besserve A., Deleglise A.-S.J., Payet M., Thouvenot E., Souberbielle J.C. (2019). Cholecalciferol in relapsing-remitting MS: A randomized clinical trial (CHOLINE). Neurol. Neuroimmunol. Neuroinflamm..

[63] Hupperts R., Smolders J., Vieth R., Holmøy T., Marhardt K., Schluep M., Killestein J., Barkhof F., Beelke M., Grimaldi L.M.E. (2019). Randomized trial of daily high-dose vitamin D3 in patients with RRMS receiving subcutaneous interferon β-1a. Neurology.

[64] Soilu-Hänninen M., Aivo J., Lindström B.M., Elovaara I., Sumelahti M.L., Färkkilä M., Tienari P., Atula S., Sarasoja T., Herrala L. (2012). A randomised, double blind, placebo controlled trial with vitamin D3 as an add on treatment to interferon-1b in patients with multiple sclerosis. J. Neurol. Neurosurg. Psychiatry.

[65] Derakhshandi H., Etemadifar M., Feizi A., Abtahi S.-H., Minagar A., Abtahi M.-A., Abtahi Z.-A., Dehghani A., Sajjadi S., Tabrizi N. (2013). Preventive effect of vitamin D3 supplementation on conversion of optic neuritis to clinically definite multiple sclerosis: A double blind, randomized, placebo-controlled pilot clinical trial. Acta Neurol. Belg..

[66] O’Connell K., Sulaimani J., Basdeo S.A., Kinsella K., Jordan S., Kenny O., Kelly S.B., Murphy D., Heffernan E., Killeen R.P. (2017). Effects of vitamin D in clinically isolated syndrome and healthy control participants: A double-blind randomised controlled trial. Mult. Scler. J. Exp. Transl. Clin..

[67] Stein M.S., Liu Y., Gray O.M., Baker J.E., Kolbe S.C., Ditchfield M.R., Egan G.F., Mitchell P.J., Harrison L.C., Butzkueven H. (2011). A randomized trial of high-dose vitamin D2 in relapsing-remitting multiple sclerosis. Neurology.

