# Supplementary material for: Radiological Benefits of Vitamin D Status and Supplementation in Patients with MS—A Two-Year Prospective Observational Cohort Study
Source: Nutrients. 2023 Mar 17;15(6):1465. doi: 10.3390/nu15061465 (PMC10052720; doi:10.3390/nu15061465)
Supplement: Supplementary file 1 [file nutrients-15-01465-s001.zip › nutrients-2260556-supplementary.pdf]

Vitamin D supplementation and factors influencing vitamin D status questionnaire

Part 1. Demographic Features

1. Education:
   - o Primary   o Further   o Higher
   - o

2. Skin phototypes:
- o Type I – ivory white in colour, burns easily, never tans
- o Type II – white, burns easily, tans minimally with difficulty
- o Type III – white, burns moderately, tans moderately
- o Type IV – beige or olive, burns minimally, tans moderately and easily
- o Type V – moderately brown, rarely burns, tans profusely
- o Type VI – dark brown or black, never burns, tans profusely

3. Heigh: _______cm
4. Weight: _______kg

Part 2. Vitamin D Supplementation

1. Are you supplementing vitamin D?
   - o Yes
   - o No (skip ahead to question 7.)

2. How long have you been supplementing vitamin D?
   - o 1 month
   - o 2-3 months
   - o 4-6 months
   - o Over 6 months

3. What kind of supplementation do you take?
   - o Cholecalciferol
   - o Alfacalcidol
   - o Others: ________________

4. How do you supplement vitamin D?
   - o Orally
   - o Subcutaneous
   - o Intramuscular
   - o Other: ________________

5. What is your supplementation dose?
   - o 500 IU
   - o 1000 IU
   - o 2000 IU
   - o 4000 IU
   - o >4000 IU

6. Why are you supplementing vitamin D?
   - o Doctor's recommendation
   - o Dietitians' recommendation
   - o My own decision about my health
   - o I'm afraid of having vitamin D deficiency

- I follow the current recommendation
- Family/friends advise
- Others: ______________
-

7. Are taking steroids?
   - Yes
   - No

8. Do you spend your time outside in spring and summer?
   - Yes
   - No, skip ahead to question 12.

9. How often do you spend your time outside?
   - Once a month
   - Once a fortnight
   - Once a week
   - Several times in a week
   - Every day
   -

10. How much time do you spend outside a day?
    - 5-15 min
    - 15-30 min
    - 30 min – 1h
    - >1h
    -

11. Do you tan/sunbathe?
    - Yes
    - No

12. Do you use sunscreen when going outside?
    - Yes
    - No, skip ahead to question 14.

13. What sunscreen do you use?
    - SPF <20
    - SPF 20-30
    - SPF 30-50
    - SPF >50
    -

14. How often do you eat following food products:

    Oily sea fish?
    - Once a month
    - Once a fortnight
    - Once a week
    - Several times in a week
    - Every day

    Red meat?
    - Once a month
    - Once a fortnight
    - Once a week
    - Several times in a week
    - Every day

    Egg yolks?
    - Once a month
    - Once a fortnight
    - Once a week
    - Several times in a week
    - Every day

    Yellow chesse?
    - Once a month
    - Once a fortnight
    - Once a week
    - Several times in a week
    - Every day

15. Do you have any comorbidities (more than one illness at a time)? Please list them:

    ____

16. Do you smoke?
    - Yes
    - No

17. If you are not supplementing vitamin D, please explain why:

- Vitamin D supplements are too expensive
- I don't believe vitamin D would be effective in my condition
- My doctor did not recommend vitamin D supplementation
- I forget to take vitamin D
- I sunbathe/tan often, I don't need supplementation
- I take too many different medications
- Others: ____________________________________
